# Advances of Fluorescent Nanodiamond Platforms for Intracellular and On-Chip Biosensing

**DOI:** 10.3390/bios14070340

**Published:** 2024-07-12

**Authors:** Taisuke Shimada, Yasuyuki Ueda, Yoshinobu Baba, Hiroshi Yukawa

**Affiliations:** 1 Institute for Quantum Life Science, National Institutes for Quantum Science and Technology (QST), Anagawa 4-9-1, Inage-ku, Chiba 263-8555, Japan; ueda.yasuyuki@qst.go.jp (Y.U.); babaymtt@chembio.nagoya-u.ac.jp (Y.B.); 2 Research Institute for Quantum and Chemical Innovation, Institutes of Innovation for Future Society, Nagoya University, Furo-cho, Chikusa-ku, Nagoya 464-8603, Japan; 3 Development of Quantum-Nano Cancer Photoimmunotherapy for Clinical Application of Refractory Cancer, Nagoya University, Tsurumai 65, Showa-ku, Nagoya 466-8550, Japan; 4 Nagoya University Institute for Advanced Research, Advanced Analytical and Diagnostic Imaging Center (AADIC)/Medical Engineering Unit (MEU), B3 Unit, Tsurumai-cho 65, Showa-ku, Nagoya 466-8550, Japan; 5 Department of Quantum Life Science, Graduate School of Science, Chiba University, 1-33 Yayoi-cho, Inage-ku, Chiba 263-8522, Japan

**Keywords:** fluorescent nanodiamonds, biosensing, surface functionalization, intracellular biosensing, lab-on-a-chip

## Abstract

Intracellular and extracellular sensing of physical and chemical variables is important for disease diagnosis and the understanding of cellular biology. Optical sensing utilizing fluorescent nanodiamonds (FNDs) is promising for probing intracellular and extracellular variables owing to their biocompatibility, photostability, and sensitivity to physicochemical quantities. Based on the potential of FNDs, we outlined the optical properties, biocompatibility, surface chemistry of FNDs and their applications in intracellular biosensing. This review also introduces biosensing platforms that combine FNDs and lab-on-a-chip approaches to control the extracellular environment and improve sample/reagent handling and sensing performance.

## 1. Introduction

Intracellular and extracellular sensing of physical and chemical variables is important for disease diagnosis and the understanding of cellular biology. The physical and chemical variables within a cell (e.g., temperature, radical species, and pH) regulate physiological process [1,2,3,4]; therefore, sensing such variables can provide essential information for a deeper understanding of cell biology [5,6]. Conversely, biomolecules in the extracellular environment are indicators of cellular state and activity, and sensing chemical variables in the extracellular environment plays a key role in disease diagnostics [7,8]. Much effort has been devoted to developing techniques for intracellular and extracellular sensing of physical and chemical variables. However, developing a sensing technique that meets the requirements of sensitivity, spatial resolution, biocompatibility, and measurement time remains challenging.

Optical sensing using fluorescent molecules and nanoparticles has the potential to satisfy above-mentioned requirements [9,10,11]. Optical sensing is characterized by high spatial resolution and noncontact measurements. The excellent photostability and brightness of fluorescent nanoparticles are particularly useful for the long-term and sensitive sensing of physical and chemical variables. Optical sensing approaches utilizing upconversion nanoparticles, quantum dots, and fluorescent nanodiamonds (FNDs) have been proposed, and FNDs are the leading candidates for optical sensing [12,13,14,15,16]. The spin-dependent optical properties of FNDs enable them to serve as excellent fluorescent reporters and sensors for various physical and chemical variables [17,18,19,20]. Moreover, the outstanding photostability, excellent biocompatibility, and rich surface chemistry of the FNDs make them suitable for intracellular and extracellular sensing [21,22,23].

Based on the potential of FNDs, this review introduces recent advances of FND platforms for intracellular and on-chip biosensing. First, we outlined the optical properties, biocompatibility, and surface chemistry of FNDs and their applications in intracellular biosensing. Although some review papers are available [14,15], the advantages of FNDs from a nanomaterial perspective and their application in intracellular biosensing are fundamental and important for discussing on-chip biosensing platforms. This review provides a novel summary of on-chip biosensing platforms that combine FNDs and lab-on-a-chip approaches to control extracellular environments and improve sample/reagent handling and sensing performance. We envision that such on-chip biosensing platforms will enhance the potential of FNDs and contribute to deepening the understanding of cell biology and developing point-of-care diagnostic tools.

## 2. The Optical Properties, Biocompatibility, and Surface Chemistry of FNDs

FNDs containing nitrogen vacancy (NV) centers (hereafter, unless otherwise specified, FNDs refer to FNDs containing NV centers) are nanoscale diamond crystals, and their excellent optical properties and rich surface chemistry are promising for biosensing applications. As various review articles are available [15,24], this section briefly introduces the optical properties, biocompatibility, and surface chemistry of FNDs. 

### 2.1. The Optical Properties of FNDs

Chemical impurities and structural defects in diamond crystals are well known to present unique optical properties, and negatively-charged NV (NV^−^) centers in diamond nanocrystals attract much attention for biosensing. The NV^−^ centers are substitutional nitrogen atoms adjacent to lattice vacancies in the diamond nanocrystals, and present two outstanding optical properties. First, the NV^−^ centers can be excited by 450~640 nm light, and emit near-infrared fluorescence (650~800 nm) without photobleaching and photo blinking [25]. The second is spin-dependent fluorescence, which enables the sensing of various physical and chemical variables using the optically detectable magnetic resonance (ODMR) concept [26]. These optical properties of NV^−^ centers promote applications of FNDs to biosensing. 

Two measurement techniques are typically employed to determine the physical and chemical variables using FNDs [13]. The first technique optically detects the energy of NV^−^ centers for spin-sublevel transition, namely the magnetic resonance frequency, by irradiating electromagnetic waves with swept frequencies [27]. The triplet ground state of NV^−^ centers is divided into three spin sublevels (ms = 0 and ms = ±1), and NV^−^ centers at the ms = 0 spin sublevel emits brighter fluorescence than those at the ms = ±1 spin sublevel. Thus, the magnetic resonance frequency of NV^−^ centers is optically detectable from fluorescence intensity fluctuations when the frequency of electromagnetic waves is swept. The magnetic resonance frequency of NV^−^ centers show the dependency on temperature of surrounding environments, and thus, this technique can optically sense temperature from the shift of the magnetic resonance frequency. The second technique utilizes optically measuring spin relaxation time of NV^−^ centers (T1 measurement), and provides information on the level of magnetic noises generated by paramagnetic species [28]. Such a technique initially manipulates the spin sublevel of NV^−^ centers by laser irradiation. After the laser switches off, monitor temporal changes in fluorescence intensity of the NV^−^ centers due to the transition of the spin sublevel. The spin relaxation time of the NV^−^ centers depends on the level of magnetic noises, so this technique can detect paramagnetic species.

### 2.2. Biocompatibility of FNDs

Biocompatibility of FNDs is important for their biological applications, particularly in biosensing within living cells. It is pointed out that the biocompatibility of FNDs originates from the chemical inertness of diamond nanocrystals and no releases of toxic chemicals from the nanocrystals [25]. Metabolic activity assays revealed that the cellular uptake of diamond nanocrystals did not significantly affect the viability of cells, such as cell lines [25], macrophages [29], and stem cells [30]. In addition to cellular viability, diamond nanocrystals have been characterized as much less genotoxic than other carbon nanomaterials (carbon nanotubes) to embryonic stem cells that are sensitive to DNA damage [31]. Moreover, the internalization of diamond nanocrystals was confirmed to cause no detectable changes in cellular morphology or protein expression levels during the differentiation process of neural cells [32]. These studies indicated that diamond nanocrystals are biocompatible and promote the application of FNDs in intracellular biosensing.

### 2.3. Chemistry of FNDs for Surface Homogenization

**Figure 1:**
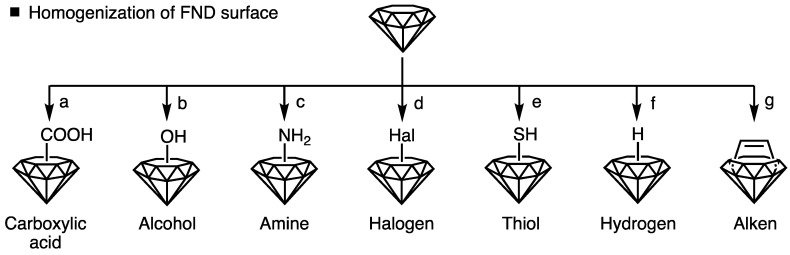
Chemistry for Homogenization of FND surfaces. (**a**) Carboxylation, (**b**) hydroxylation, (**c**) amination, (**d**) halogenation, (**e**) thiolation, (**f**) hydrogenation, and (**g**) thermal annealing for terminating with unsaturated sp^2^ carbons (alken).

Surface homogenization is fundamental for the stable dispersion of FNDs in biologically relevant media and functionalization of FND surfaces. This is because pristine FND surfaces have complex structures with a mixture of sp^2^ and sp^3^ carbon atoms, and the purity, surface composition, and reactivity of FND surfaces vary widely [33]. Surface homogenization typically involves two steps: the removal of surface impurities and tailoring of surface functional groups. First, graphitic and amorphous carbon on the FND surfaces are removed using a high-temperature air-oxidation approach [34]. FND surfaces are terminated with carbon-oxygen-containing functional groups via the oxidation approach, and these groups are useful for tailoring the surface functional groups. Next, the FND surfaces with carbon–oxygen-containing functional groups were tailored to the desired functional groups (Figure 1). Hereafter, the details of the tailoring of the surface functional groups are introduced.

First, tailoring FND surfaces with carboxyl groups requires acid or ozone treatment after air oxidation (Figure 1a). Acid treatments included mixed acids (a mixture of nitric and sulfuric acids) and hydrogen peroxide/sodium hydroxide [35,36,37]. In addition to terminating the FND surfaces with carboxyl groups, acid or ozone treatments can improve the purity and aqueous dispersibility of FNDs by removing surface impurities. Tailoring FND surfaces with hydroxyl groups is also common because hydroxyl groups increase the aqueous dispersibility of FNDs and the availability of chemical reaction routes (Figure 1b). The major approaches for hydroxylating FND surfaces include the Fenton reaction (a mixture of hydrogen peroxide and iron (II) sulfate) and photochemical reactions [38,39,40,41]. Third, terminating the FND surfaces with amino groups renders the surface cationic, hydrophilic, and applicable to various bioconjugations (Figure 1c). Various approaches for amine group formation are available—for example, the reaction of hydroxylated FND surfaces with (3-aminopropyl) trimethoxysilane [42]. Fourth, halogenated FND surfaces are useful for synthetically immobilizing desired functional molecules owing to their high reactivity with various nucleophilic reagents (Figure 1d). The major approaches for halogenating FND surfaces include chemical treatments using fluorine and hydrogen fluoride [43]. Fifth, thiolated FND surfaces are important for selectively immobilizing the desired biomolecules with a controlled number and position of reaction sites (Figure 1e). This is because, in addition to the reactivity of sulfhydryl groups, proteins, antibodies, and other biomolecules contain sulfhydryl groups, and their number is much lower than that of the carboxyl and amine groups. Approaches for tailoring FND surfaces with sulfhydryl groups include photochemical reactions using elemental sulfur and carbon disulfide [44]. Sixth, the tailored FND surfaces with hydrogen groups provide reaction sites for the formation of strong carbon–carbon single bonds that are resistant to oxidation and hydrolysis (Figure 1f) [42]. The hydrogenated FND surfaces were processed via thermal annealing in a hydrogen atmosphere [45]. Finally, the FND surfaces with unsaturated sp^2^ carbons (alkenes) were formed via thermal annealing. The reactivity of FND surfaces is useful for cycloaddition reactions (Figure 1g) [46]. Homogenized surfaces with functional groups enable FNDs to stably disperse in biologically relevant media and/or immobilize the desired functional molecules on the FND surfaces; thus, the chemistry of FNDs for surface homogenization is important for their biological applications.

## 3. FND Platforms for Intracellular Biosensing

FND-based intracellular biosensing can provide information on various physical and chemical variables (e.g., temperature, electric field, radical species, and pH) in the local environment. Temperature and radical species are especially important for the cellular state and function [9,47], so studies on the intracellular sensing of temperature [48,49] and radical species [20,50,51] have been actively conducted. pH is also a crucial parameter of the intracellular environment; however, recent studies have only succeeded in FND-based pH sensing under biologically relevant conditions [18,52], so pH sensing in living cells remains challenging [15]. In this section, we introduce the applications of FNDs for intracellular sensing of temperature and radical species.

### 3.1. Sensing of Intracellular Temperature

Intracellular temperature is a possible factor that modulates biophysical and biochemical processes within a cell via various biochemical reactions; thus, temperature sensing inside a cell is key to deepening our understanding of intracellular temperature during biological processes.

**Figure 2:**
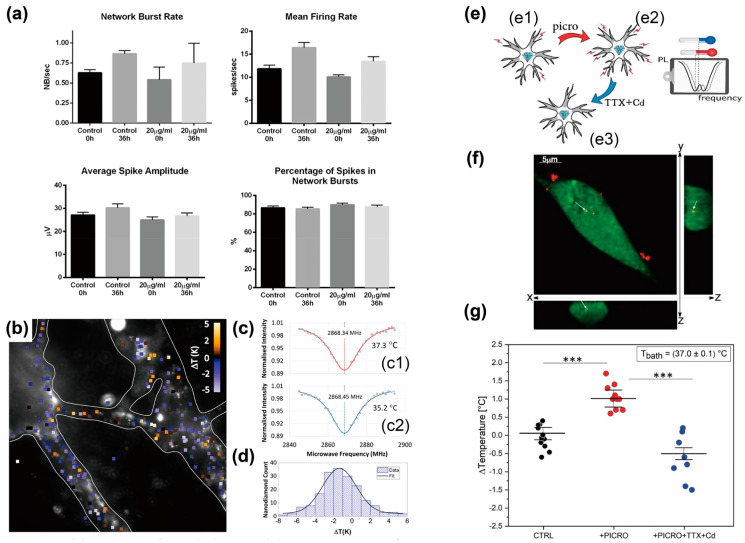
FNDs-based Intracellular temperature measurements. (**a**–**c**) Intracellular temperature mapping using non-neurotoxic FNDs. (**a**) Neuronal activity parameter comparison between control (non-FNDs) and FNDs-treated neurons. The parameters are network burst rate, mean firing rate, average spike, amplitude, and percentage of spikes in the network bursts. (**b**) Intracellular temperature mapping of primary cortical neurons using FNDs. The image was captured at 37.3 °C. (**c**) Typical ODMR spectra captured at (**c1**) 37.3 °C and (**c2**) 35.2 °C. (**d**) Histogram of temperature fluctuations measured from 255 FNDs. Reprinted with permission from [54]. (**e**–**g**) Detection of neural activities-induced intracellular temperature elevations using FNDs. (**e**) Schematics depicting FNDs-based temperature measurements (**e1**) under control (CTRL) conditions, (**e2**) after chemical potentiation of neural activities by picrotoxin (picro), and (**e3**) after chemical inhibition of neural activities by tetrodotoxin and cadmium chloride (TTX+Cd). (**f**) Confocal fluorescent images of hippocampal neurons treated with FNDs. Green, cytoplasm; red, FNDs. White arrows represent a FND nanoparticle internalized within a cell. (**g**) Plots of intracellular temperature fluctuations measured by FNDs. The asterisks indicate a statistical difference (***, *p* < 0.0001). The measurements were conducted as illustrated in Figure 2e. Reprinted with permission from [55].

FND-based intracellular temperature sensing has been applied to various cell types, including stem cells and neurons, to investigate the relationship between intracellular temperature and cellular functions. FND-based temperature sensing within stem cells shows no significant cytotoxicity [53]. FNDs were internalized into adipose-derived stem cells by culturing the cells in a medium containing FNDs for 24 h. FNDs presented low cytotoxicity to the stem cells incubated with FNDs at the concentration of less than 500 μg/mL and had no significant effects on the ability of the cells to secrete various growth factors and differentiate into adipocytes and osteoblasts. The magnetic resonance frequency of FNDs within a cell was confirmed to shift in a temperature-dependent manner via the ODMR concept; thus, FND-based temperature sensing could provide the precise intracellular temperature of stem cells. The biocompatibility and temperature sensitivity of FNDs are key for intracellular temperature sensing and could play an important role in probing the relationship between intracellular temperature and cellular functions. Thereafter, FND-based temperature sensing was utilized for intraneuronal temperature mapping (Figure 2a–d) [54]. Electrophysiological monitoring of neuronal cell activity (e.g., network burst rate, mean firing rate, and average spike) revealed that FNDs internalized by primary cortical neurons had a slight effect on their activity over various periods. Simultaneous ODMR measurements of thousands of FNDs within neuronal cells have facilitated the mapping of intracellular temperatures in a neuronal cell network. This is an important example in which FND-based temperature sensing can provide intracellular temperature with spatial information without significantly affecting cellular activities. Moreover, FND-based temperature sensing revealed elevated intracellular temperatures in response to neural activity (Figure 2e–g) [55]. FNDs were internalized by hippocampal neurons, and the neural activity of the cells were potentiated and inhibited by chemical stimulation. FNDs-based temperature sensing reported 1 °C of intracellular temperature fluctuation between the chemically potentiated and inhibited cells. FND-based temperature sensing requires FND internalization and laser irradiation for ODMR measurements, and these experimental procedures were supplementally confirmed not to induce any detectable inhibition of neural activity. This demonstrates that FND-based temperature sensing can detect intracellular temperature fluctuations originating from cellular activity. In summary, these three examples highlight that nanoscale temperature sensing using FNDs is a promising tool for elucidating the relationship between the intracellular temperature and cellular function. 

**Figure 3:**
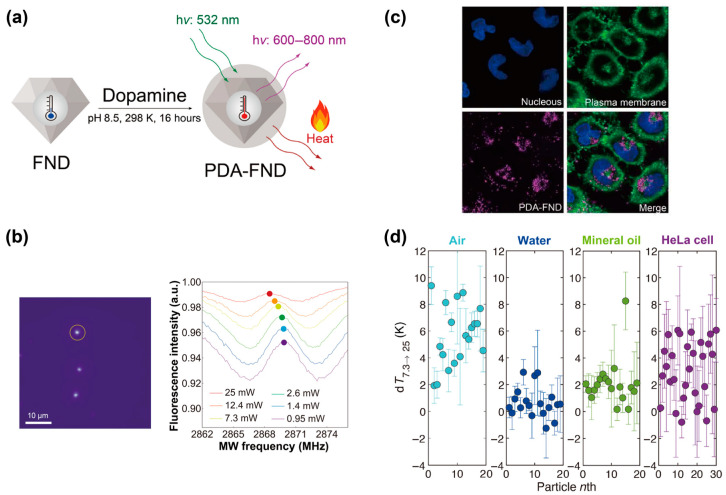
In situ measurements of intracellular thermal conductivity using PDA-FNDs. (**a**) A schematic illustration of a PDA-FND. PDA and FND served as a light-induced nanoheater and nanothermometer, respectively. (**b**) A typical fluorescent image and ODMR spectra of PDA-FNDs. The ODMR spectra was obtained from a PDA-FND indicated by the yellow circle in the image. The ODMR spectra of the PDA-FND were recorded with various excitation laser powers. (**c**) Confocal fluorescent images of HeLa cells treated by PDA-FNDs. Image size, 92 µm × 92 µm. (**d**) Plots of temperature fluctuations measured in various environments. d*T*_7.3→25_ means the temperature fluctuation when the excitation laser power was changed from 7.3 mW to 25 mW. Reprinted with permission from [57].

Two examples of the combination of intracellular temperature sensing with precise temperature control have been reported to probe thermal properties within a single cell, including the temperature gradient and thermal conductivity. Pioneering work has demonstrated the sensing of intracellular temperature at the nanometer scale to map the temperature gradient within living cells [56]. FNDs and gold nanoparticles (AuNPs) served as nanothermometers and nanoheaters, respectively, and were internalized into human fibroblasts via nanowire-assisted intracellular delivery. FNDs at different distances from the AuNPs within the cell were used, and the AuNPs were irradiated with a green laser to generate heat. FND-based temperature sensing provides information on local intracellular temperatures against the distance between each FND and AuNP. The FND with a shorter distance from the AuNPs exhibited a higher local temperature than those with a longer distance from the AuNPs. The highlight of this study is that the combination of FND-based temperature sensing and nanoheaters revealed that living cells have an intracellular temperature gradient. Another example utilized polydopamine (PDA)-coated FNDs (PDA-FNDs) to measure intracellular thermal conductivity (Figure 3) [57]. The FND surfaces were coated with a PDA layer by polymerizing dopamine under basic conditions, and the FNDs and PDA served as nanothermometers and nanoheaters, respectively. PDA-FNDs facilitated the generation of heat by irradiating the 532 nm laser and measuring the temperature via the ODMR concept. HeLa cells internalized the PDA-FNDs with no detectable cytotoxicity, and the PDA-FNDs within the cells enabled the measurement of optically induced intracellular temperature fluctuations. The measurements using the PDA-FNDs determined the intracellular thermal conductivity as 0.11 ± 0.04 W m^−1^ K^−1^, which was significantly lower than that of water. This demonstration shows that the PDA-FNDs, serving as nanothermometers and nanoheaters, allow thermal conductivity measurements with nanoscale spatial resolution. In summary, these two examples suggest the potential of FND-based nanoheater/nanothermometer composites as tools for probing intracellular thermal properties associated with heat dissipation processes.

### 3.2. Sensing of Intracellular Free Radical

Free radicals are important intracellular molecules that act as stress indicators [58]. However, the detection of free radicals within cells remains a challenge because they are short-lived and highly reactive [9]. NV centers in diamond nanocrystals exhibit excellent sensitivity to spin noise induced by free radicals; thus, FNDs are promising candidates for the local sensing of free radicals within a cell [28]. This section provides four exemplified studies on the FND-based sensing of free radicals under biologically relevant conditions and within a cell.

**Figure 4:**
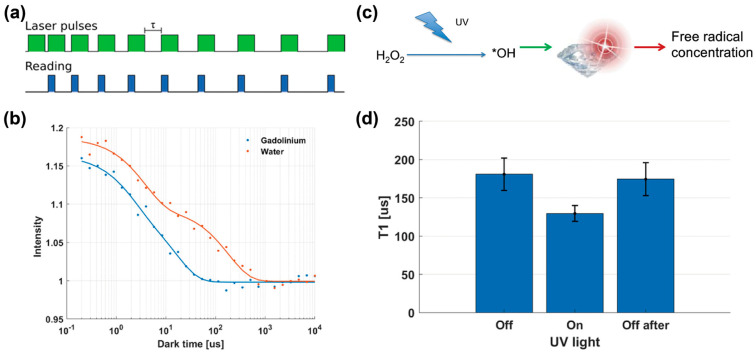
Real-time detection of free radicals using FNDs. (**a**) A schematic depicting pulsing sequence for FND-based T1 measurements. (**b**) Typical T1 curves measured in water with and without 0.5 mM Gd^3+^. (**c**) A schematic showing real-time detection of UV-induced free radical generations. (**d**) FND-based T1 measurements for detecting free radicals. The decrease of T1 relaxation time under UV irradiation corresponds 0.9 μM hydroxyl radicals was generated. Reprinted with permission from [59].

A study on T1 measurements using FNDs was conducted to verify whether the detection of spin noise under biologically relevant conditions and real-time detection of free radicals were possible (Figure 4) [59]. T1 measurements using FNDs were performed in water, phosphate-buffered saline (PBS), and cell culture medium. Gd^3+^, a commonly used contrast agent, was used as the test sample. T1 measurements for FNDs in water and PBS provided comparable T1 relaxation times, whereas T1 measurements for FNDs in the medium offered a shorter T1 relaxation time than those for FNDs in water and PBS owing to protein adsorption on the FND surfaces. Thereafter, the FNDs were utilized for the real-time detection of free radicals generated by photolysis, because the photolysis of hydrogen peroxide is a biologically important reaction. T1 measurements for the FNDs before, under, and after ultraviolet (UV) irradiation showed a reduced T1 relaxation time when hydrogen peroxide was irradiated with UV radiation, indicating that the FNDs could detect free radicals generated by photolysis in real time. Moreover, the photostability of FNDs allows for the repeated measurement of free radicals with nanoscale spatial resolution without photobleaching. In summary, the verification of FND-based T1 measurements under biologically relevant conditions provides fundamental insights for free-radical sensing in living cells and organisms.

**Figure 5:**
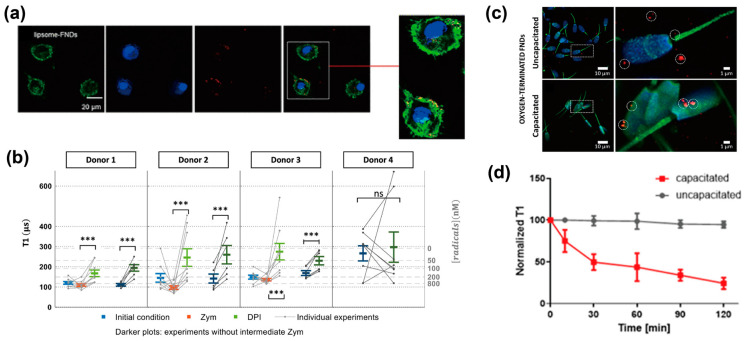
FND-based T1 measurement of intracellular free radicals. (**a**,**b**) Free radical detection in primary human DCs using liposome-coated FNDs. (**a**) Fluorescent images of primary human DCs labeled with liposome-coated FNDs. Green, F actin filaments; blue, nuclei; red, FNDs. (**b**) T1 measurements for detecting free radical generation in single primary human DC. The T1 value was initially measured in single DCs, and then, T1 measurement was performed after the single cells were treated by zymosan (Zym, chemical activator of NADPH oxidase). Finally, the T1 value was collected from single DCs treated by diphenyleneiodonium chloride (DPI, chemical inhibitor of NADPH oxidase). The asterisks indicate a significant difference (***, *p* < 0.0001), and ns means no significant difference. Reprinted with permission from Ref. [61]. (**c**,**d**) FNDs-based detection of free radicals in single sperm cells. (**c**) Confocal fluorescent images of uncapacitated and capacitated sperm cells labeled with oxygen terminated FNDs. Green, F actin filaments; blue, nuclei; red, FNDs. The circles indicate a few FNDs to give an example. (**d**) Plots of T1 response during sperm capacitation. Y-axis means normalized T1. Reprinted with permission from Ref. [62].

Thereafter, three studies demonstrating free radical detection within cells were performed. First, FNDs internalized by J774 macrophages were used to detect intracellular free radicals, including NO* [60]. A time series of T1 measurements using intracellular FNDs revealed a change in the T1 relaxation time when macrophages were chemically stimulated by NO* production inhibitors. The results of FND-based monitoring of NO* production at the single-cell level were supported by free radical detection using a conventional fluorescent probe, demonstrating the ability of FNDs to sense NO* production within a cell. This monitoring revealed that individual macrophages exhibited heterogeneity in the dynamics of NO* production in response to chemical stimulation. Second, free radical detection using liposome-coated FNDs was demonstrated in primary human dendritic cells (DCs; Figure 5a,b) [61]. DCs from donors have different endocytosis and free radical production abilities than cell lines, so the utilization of FND-based free radical sensing in primary cells is biologically and clinically important. The uptake of FNDs by primary DCs was assisted by a liposome coating with no significant cytotoxicity, and the internalized FNDs were confirmed to localize to the endophagosome. T1 measurements utilizing intracellular FNDs enabled the quantification of free radical production by nicotinamide adenine dinucleotide phosphate (NADPH) oxidase within cells. Moreover, T1 measurements revealed that the free radical concentration within a cell fluctuated due to chemical potentiation and inhibition of intracellular NADPH oxidase, and DCs showed heterogeneity in free radical production between donors. Finally, FND-based sensing of free radicals in single sperm cells was performed to identify the source of free radicals during sperm maturation (Figure 5c,d) [62]. FNDs with oxygen-terminated surfaces effectively labeled sperm cells and labeling sperm cells with FNDs did not reduce cell activity or viability. T1 measurements using FNDs with oxygen-terminated surfaces enabled the local detection of free radicals in sperm cells. These measurements revealed altered concentrations of localized free radicals in the capacitated and uncapacitated sperm cells. A combination of FND-based T1 measurements and chemical inhibition of free radical production has contributed to the identification of the source of free radicals during sperm capacitation. These examples emphasize that the advantages of FND-based free-radical sensing are real-time, continuous, and high spatial resolution compared to conventional spin labels and fluorescent probes. Thus, nanoscale detection of free radicals using FNDs is a powerful tool for revealing the roles played by free radicals during biological processes.

## 4. FNDs-Based On-Chip Biosensing Platforms

Although FND-based biosensing is useful for probing biological processes, it remains in its infancy. For example, FND-based biosensing with the controllability of extracellular environments, enhanced reproducibility, and high throughput is necessary to probe intracellular environments in detail. In addition, owing to the promising spin-dependent optical properties of FNDs, the development of FND-based biosensors for bacteria and viruses, in addition to mammalian cells, is important in microbiology and diagnostics [63]. One way to develop an FND-based biosensing platform is to combine lab-on-a-chip approaches [64], which are characterized by a reduced sample/reagent volume, enhanced reproducibility, high throughput, and precise controllability of samples and reagents [65]. This section focuses on the combination of lab-on-a-chip approaches and FND-based biosensing, and outlines their applications, from on-chip intracellular and extracellular sensing to spin-enhanced virus detection.

### 4.1. FNDs-Based On-Chip Biosensing with Enhanced Reproducibility and Fluidic Controllability

As the lab-on-a-chip approach facilitates the local manipulation of fluid and electromagnetic fields, FND-based on-chip biosensing has the potential to meet the requirements of controllability of the extracellular environment, enhanced reproducibility, and high throughput. In this paper, we introduce two examples of FND-based on-chip biosensing studies.

**Figure 6:**
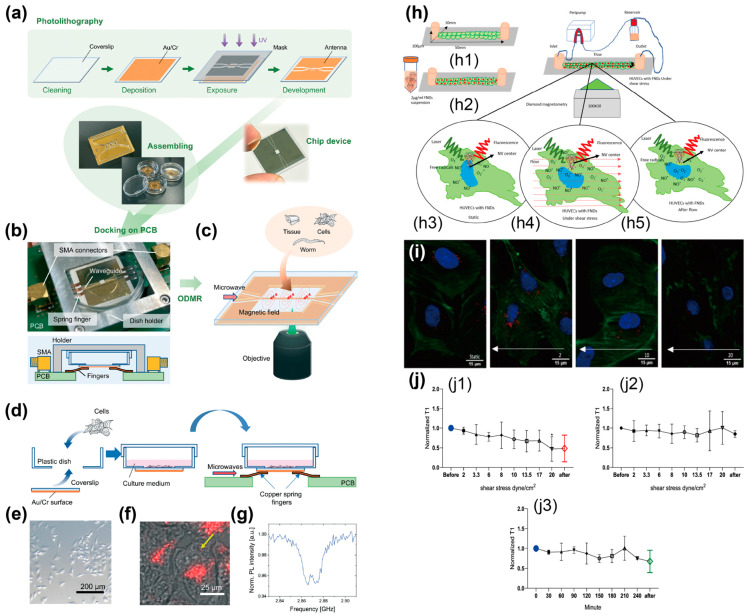
FNDs-based on-chip biosensing with enhanced reproducibility and fluidic controllability. (**a**–**g**) A notch-shaped coplanar antenna structure for spatially uniform microwave irradiation. (**a**) Schematics depicting fabrication process of a chip device with the coplanar antenna. (**b**) A schematic and photo of the chip device docked with a dish and a printed circuit board (PCB). (**c**) A schematic showing microwave irradiation to biological samples via the coplanar antenna. (**d**) Schematics depicting cell culture on the chip device and ODMR measurement. (**e**) A photo of living HeLa cells cultured on the chip device. (**f**) A merged bright-field image with fluorescence of intracellular FNDs. (**g**) An ODMR spectra that was captured from a yellow arrow in Figure 6f. Reprinted with permission from [66]. (**h**–**j**) Real-time detection of intracellular free-radicals inside a microchannel. (**h**) Schematics depicting (**h1**) HUVEC culture, (**h2**) FND uptakes, and (**h3**–**h5**) free-radical detection (**h3**) before flow, (**h4**) under shear stress, (**h5**) after flow. (**i**) Confocal fluorescent images of FND-labeled HUVECs inside a microchannel with and without shear stress. (**j**) T1 curves of FNDs with and without HUVECs under the shear stress conditions. (**j1**) FNDs inside HUVECs and (**j2**) FNDs in a microchannel were measured under the flow rate from 2 to 20 dyne/cm^2^. (**j3**) FNDs inside HUVECs were measured under the flow rate from 4 dyne/cm^2^ for 4 h. Reprinted with permission from [67].

FND-based biosensing with enhanced reproducibility requires uniform microwave irradiation for the ODMR concept, so an antenna structure for guiding microwaves is key to realizing on-chip biosensing. However, the miniaturization of chip-integrable antenna structures remains challenging owing to the limitations of broadband and spatially uniform microwave irradiation. A notch-shaped coplanar antenna structure was proposed for the spatially uniform irradiation of broadband microwaves over large areas (Figure 6a–g) [66]. The antenna structure was based on gold films patterned on a glass chip using photolithography and offered a millimeter-scale sensing area. Numerical simulations support the idea that the antenna structure enables the irradiation of spatially uniform microwaves over the sensing area. The demonstration of microwave irradiation using the antenna structure showed successful acquisition of ODMR spectra from FNDs within a living HeLa cell and an organism (*Caenorhabditis elegans*) with uniform ODMR signal intensity. The advantages of this antenna structure are chip integrability and spatially uniform microwave irradiation, which facilitate the realization of on-chip biosensing platforms that can acquire spatially uniform ODMR spectra.

Another study reported on FND-based biosensing inside a microchannel. For the real-time detection of intracellular free radicals induced by shear stress, T1 measurements using FNDs were combined with a microfluidic platform (Figure 6h–j) [67]. Precise fluidic manipulation of the microfluidic platform provided shear stress to human umbilical vein endothelial cells (HUVECs), and the FNDs served to monitor the free radicals generated within single cells in real time. The fibronectin coating on the microchannel surfaces assisted in cell adhesion, and the FNDs were internalized into the cells by filling the microchannel with a solution containing FNDs. T1 measurements using FNDs revealed that cells under shear stress produced more free radicals than those under static conditions, indicating that fluid flow in the extracellular environment is an important factor for cellular stress. This study indicates that the combination of FND-based biosensing and a microfluidic platform is a promising tool for evaluating cellular responses to fluids in real-time, which will contribute to the biological understanding of cardiovascular diseases induced by shear stress.

These studies indicate that lab-on-a-chip approaches are useful to reproducibly perform FND-based intracellular biosensing under well-controlled extracellular environment. For example, FND-based intracellular sensing on a chip can probe fluctuations of intracellular environment in response to repeated chemical stimulations. Although much effort may be required, FND-based intracellular sensing on a chip will contribute to deepening the understanding of cell biology.

### 4.2. On-Chip Biosensing Utilizing FNDs Embedded in Extracellular Matrixes

Embedding FNDs in extracellular matrixes on a chip is a current trend in sensing the extracellular environment [68,69]. This section describes two studies that demonstrate the embedding of FNDs in nanofibers and deformable resin membranes.

First, embedding FNDs in poly-ε-caprolactone nanofibers was reported toward the development of a biomedical scaffold with the temperature sensing ability [70]. FNDs were added to a solution containing nanofiber prepolymers and FND-embedded nanofibers were formed via electrospinning. The biocompatibility and hydrophilicity of FNDs are key to enhancing the adhesion and proliferation of stem cells to the nanofibers, and the FND-embedded nanofibers presented no detectable cytotoxicity. Moreover, measurement of the ODMR spectrum of FNDs embedded in nanofibers was demonstrated, which promises to develop a nanofiber scaffold that can sense the extracellular temperature associated with living cells on the nanofibers.

**Figure 7:**
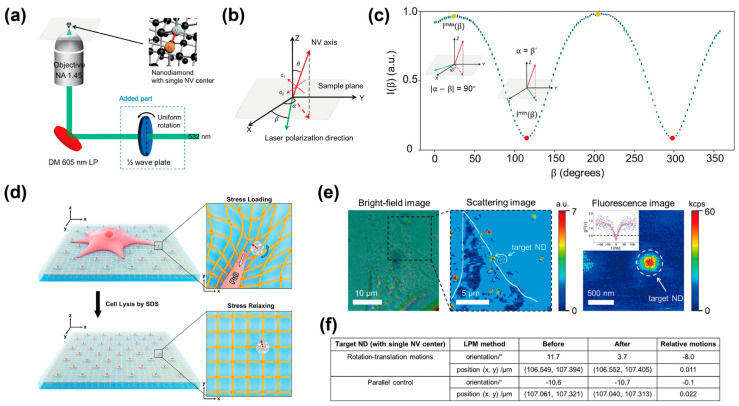
Detection of translational and rotational movements caused by cellular traction force on a deformable substrate. (**a**) A schematic diagram depicting an optical configuration for measuring one-dimensional orientation of a single NV center in FND. (**b**) A schematic illustration of the NV axis orientation (solid red arrow) with a corresponding projection line (dashed red arrow) and a laser polarization direction (solid green arrow). (**c**) A typical liner polarization modulation curve of a single NV center. The fluorescent intensities of the same NV center are plotted as a function of laser polarization direction. (**d**) Schematic illustrations depicting detection of translational and rotational movements caused by cellular traction force. The orientation and position of FNDs were measured before and after cell lysis. (**e**) Typical bright field (**left panel**) and scattering (**middle panel**) images of a cell on FNDs-casted silicon resin substrate. (**Right panel**) is a typical fluorescent image of a target FND which was indicated by a white arrow in the scattering image. (**f**) A table showing the orientation and position of the target FND before and after cell lysis. Reprinted with permission from [71].

Another example is the use of FNDs to detect translational and rotational movements caused by cellular traction forces acting on deformable substrates (Figure 7) [71]. The FNDs were cast onto a cover glass with a deformable silicon resin membrane modified with fibronectin to enhance cell adhesion. The locations and orientations of the FNDs before and after cell lysis were measured to analyze the movement of the FNDs mechanically generated by a cell on the silicon membrane. Optically polarized selective excitation of NV centers was key to measuring the location and orientation of FNDs, and the accuracy of the location and orientation was evaluated to be approximately 0.5°/7.5 s and 2 nm/min, respectively. Although cell lysis was required, a combination of the FND-cast silicon membrane and selective excitation of NV centers was effective in detecting the 8° rotation and 11 nm translation of the silicon membrane deformed by the cell traction force. This study highlights that the utilization of on-chip FNDs can monitor cell-induced translational and rotational motions of a substrate and provide mechanobiological evidence to understand how a cell moves on a substrate.

These two examples highlight that FNDs embedded in extracellular matrixes are useful for sensing physical and chemical variables outside the cells. In future, the combination of FNDs internalized in cells and embedded in matrices will provide a biosensing platform that can simultaneously monitor intracellular and extracellular variables.

### 4.3. FNDs-Based On-Chip Biosensing for Biomolecular Assays

Ultrasensitive bioassays of biomolecules, such as nucleic acids and proteins, are applications of FND-based biosensing [63]. Such bioassays typically include processes for target capture, optical labeling, and washing, which require transportation of samples and reagents. The lab-on-a-chip approach facilitates sample/reagent transportation with a reduced sample/reagent volume [72,73], so the requirement for sample/reagent transportation in bioassays is fulfilled by combining FND-based biosensing and lab-on-a-chip approach. This section introduces two studies of FND-based on-chip biosensing for ultrasensitive bioassays. 

**Figure 8:**
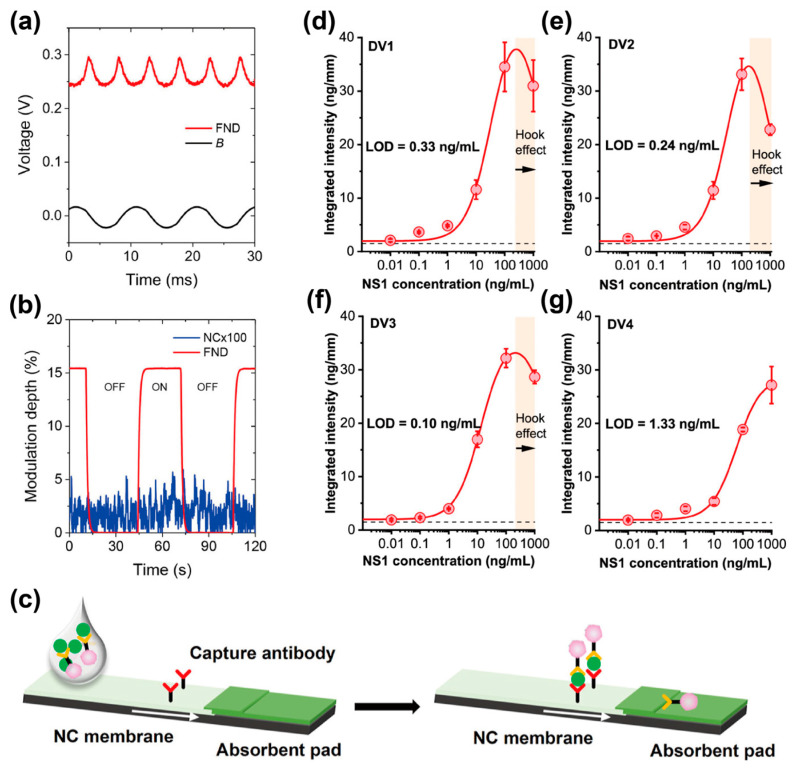
Spin-enhanced lateral flow immunoassay. (**a**,**b**) The modulated fluorescent intensity of FNDs by irradiating the alternating current (AC) magnetic field. (**a**) The fluorescent intensity of FNDs on a NC membrane under the irradiation of the AC magnetic field. The fluorescent intensity and the magnetic field were measured by a photomultiplier tube and a Gauss meter, respectively. (**b**) Time traces of modulated fluorescent intensities from FNDs and a NC membrane under the exposure to the AC magnetic field. Reprinted with permission from [76]. (**c**–**g**) Lateral flow immunoassay using FNDs. (**c**) Schematics depicting a sandwich-based lateral flow immunoassay using FNDs. FNDs were conjugated with anti-dengue antibodies via physical adsorption, and NS1 antigens were labeled with the antibodies-FND prior to flowing into a NC membrane. (**d**–**g**) Calibration curves and limit of detection (LOD) of spin-enhanced lateral flow immunoassay for assaying different serotypes of dengue virus ((**d**), DV1; (**e**), DV2; (**f**), DV3; (**g**), DV4). Reprinted with permission from [75].

The spin-dependent optical properties of FNDs have been utilized in lateral flow bioassays on paper-chip platforms for ultrasensitive fluorescent labeling [74]. The FND surfaces were modified with biomolecules that could bind to the biochemically tagged targets. After the tagged targets were captured on the paper chip, a solution containing modified FNDs was used to label them. Microwave irradiation enabled the modulation of fluorescent signals from the FNDs at a set frequency, so the fluorescent signals were selectively detected by separating them from the background signals. The modulative fluorescent signals of the FNDs on a paper-chip platform provided 10^5^ times higher sensitivity than gold nanoparticles as labeling reagents. The combination of FNDs and a paper chip was utilized in a sandwich assay for HIV-1 RNA detection, and RNA detection at the single-copy level was demonstrated using a 10-min isothermal amplification of RNA. Another study reported a bioassay for a nonstructural protein (NS1) antigen derived from the dengue virus by combining FND-based biosensing with a nitrocellulose (NC) membrane chip platform (Figure 8) [75,76]. The FND surfaces were conjugated with antibodies via physical adsorption to selectively label the NS1 antigen. A solution containing antibody-conjugated FNDs was mixed with the sample solution to label the NS1 antigen prior to flowing into the NC membrane chip, where the complex of FNDs and NS1 antigen was captured by antibodies immobilized on the NC membrane chip. The spin-dependent optical properties of FNDs enabled the separation of fluorescent signals derived from the FNDs and the NC membrane [76], and the NC membrane chip platform using FNDs showed 5000 times higher sensitivity than that using gold nanoparticles. Conjugation of FND surfaces with different antibodies allowed to differentiate the serotypes of NS1 antigen in sample solutions, and selective detection of NS1 antigen serotypes with the limit of detection from 0.1 to 1.3 ng/mL was achieved by using the paper-chip platform. In summary, paper chips can generate a fluidic flow for sample/reagent transportation via capillary force [72], and sample/reagent transportation on paper chips facilitates bioassay processes using FNDs. Thus, these examples indicate that the combination of FNDs and paper chips is an ultrasensitive bioassay platform for biomolecules, including nucleic acids and proteins, and will contribute to the development of point-of-care diagnostic tools.

## 5. Conclusions and Future Perspectives

In summary, we outlined the optical properties, biocompatibility, and surface chemistry of FNDs and their applications in intracellular and on-chip sensing. Engineering FND surfaces plays a fundamental role in enhancing their selectivity and dispersibility in biological environments. FNDs internalized into a cell can sense physical and chemical variables locally without significant cytotoxicity. Such intracellular sensing allows researchers to probe biophysical and biochemical events inside a cell. Moreover, the combination of FND-based sensing with the lab-on-a-chip approach is a direction for expanding its applications. Here, we present studies on intracellular sensing in physically and chemically controlled environments, extracellular environment sensing, and ultrasensitive bioassays.

FND-based intracellular and on-chip sensing is still in its infancy; therefore, further studies are required. Intracellular sensing has challenges in terms of sensitivity and selectivity; one example is the selective transportation of FNDs to intracellular components (e.g., organelles) for the local monitoring of biophysical and biochemical events. In contrast, owing to the limited number of studies on FND-based on-chip sensing, considerable effort is required to develop an unprecedented sensing platform by combining FND-based sensing and lab-on-a-chip approach. Despite these challenges, FND-based intracellular and on-chip sensing promises to contribute to the understanding of cell biology and development of disease diagnostic tools.

## Data Availability

No data were used for the research described in the article.
